# Sublethal Concentrations of Antibiotics Cause Shift to Anaerobic Metabolism in *Listeria monocytogenes* and Induce Phenotypes Linked to Antibiotic Tolerance

**DOI:** 10.3389/fmicb.2016.01091

**Published:** 2016-07-12

**Authors:** Gitte M. Knudsen, Arvid Fromberg, Yin Ng, Lone Gram

**Affiliations:** ^1^Department of Bioengineering, Technical University of DenmarkKongens Lyngby, Denmark; ^2^National Food Institute, Technical University of DenmarkSøborg, Denmark

**Keywords:** *Listeria monocytogenes*, sublethal antibiotic concentrations, gene expression, metabolism monocin, biofilm

## Abstract

The human pathogenic bacterium *Listeria monocytogenes* is exposed to antibiotics both during clinical treatment and in its saprophytic lifestyle. As one of the keys to successful treatment is continued antibiotic sensitivity, the purpose of this study was to determine if exposure to sublethal antibiotic concentrations would affect the bacterial physiology and induce antibiotic tolerance. Transcriptomic analyses demonstrated that each of the four antibiotics tested caused an antibiotic-specific gene expression pattern related to mode-of-action of the particular antibiotic. All four antibiotics caused the same changes in expression of several metabolic genes indicating a shift from aerobic to anaerobic metabolism and higher ethanol production. A mutant in the bifunctional acetaldehyde-CoA/alcohol dehydrogenase encoded by *lmo1634* did not have altered antibiotic tolerance. However, a mutant in *lmo1179* (*eutE*) encoding an aldehyde oxidoreductase where rerouting caused increased ethanol production was tolerant to three of four antibiotics tested. This shift in metabolism could be a survival strategy in response to antibiotics to avoid generation of ROS production from respiration by oxidation of NADH through ethanol production. The monocin locus encoding a cryptic prophage was induced by co-trimoxazole and repressed by ampicillin and gentamicin, and this correlated with an observed antibiotic-dependent biofilm formation. A monocin mutant (Δ*lmaDCBA*) had increased biofilm formation when exposed to increasing concentration of co-trimoxazole similar to the wild type, but was more tolerant to killing by co-trimoxazole and ampicillin. Thus, sublethal concentrations of antibiotics caused metabolic and physiological changes indicating that the organism is preparing to withstand lethal antibiotic concentrations.

## Introduction

*Listeria monocytogenes* is a Gram-positive bacterium that can cause listeriosis in susceptible individuals (Cossart, 2011). The disease is food-borne and although rare (2–8 cases per million per year), it causes the highest mortality among food-borne pathogens (20–30%). For instance in 2014, 41 individuals were infected in a Danish listeriosis outbreak with 17 fatalities (Kvistholm Jensen et al., 2016). *L. monocytogenes* mainly infects elderly and immunocomprised individuals, and their survival depends on successful antibiotic treatment, with the first choice often being ampicillin with or without gentamicin (Hof et al., 1997; Kvistholm Jensen et al., 2010; Cossart, 2011). Besides being a human pathogen, *L. monocytogenes* is also a saprophyte with a natural habitat in decaying plant material (Cossart, 2011). Whilst the bacterium is exposed to lethal concentrations of antibiotics during listeriosis treatment, it will likely be exposed to a window of antibiotic concentrations including low and sublethal concentrations both in the host (Hof et al., 1997) as well as in its saprophytic lifestyle due to co-existence with antibiotic-producing microorganisms (Andersson and Hughes, 2014; Mitosch and Bollenbach, 2014) and even extremely low antibiotic concentrations can select for maintenance of multi-resistance plasmids and resistant bacteria (Gullberg et al., 2011, 2014).

*Listeria monocytogenes* is susceptible to most antibiotics but most antibiotics are bacteriostatic and not bactericidal against *L. monocytogenes* (Hof et al., 1997; Hof, 2003). It is intrinsically resistant to nalidixic acid, fosfomycin and third generation cephalosporins (Hof, 2003) and the level of acquired antibiotic resistance is low in *L. monocytogenes* as compared to other pathogens such as *Staphylococcus aureus* and *Pseudomonas aeruginosa*; however, antibiotic resistance in *L. monocytogenes* is slowly rising (Granier et al., 2011). Acquisition of antibiotic resistance by point mutations could be a slow process, as *L. monocytogenes* has a low mutation rate leading to a very stable genome and high percentage of core genes (Kuenne et al., 2013). However, the presence of known antibiotic resistance cassettes is also low in *L. monocytogenes.* Reflecting on the saprophytic lifestyle of this organism and the likely constant or repeated exposure to antibiotic producing microorganisms, we hypothesize that *L. monocytogenes* may have adapted similar modes to respond to antibiotics with that is found in other bacteria (Bernier and Surette, 2013).

The bacteriostatic/bactericidal action of antibiotics has, due to the tremendous success in combating infections, been the prime focus of antibiotic research. However, antibiotics also appear to have an ecological and/or metabolic role and Andersson and Hughes (2014) suggested that in antibiotic-producing organisms antibiotic eliminate excess reducing power and thereby play a metabolic role. Other studies have proposed that they act as signals or cues for gene expression causing changes in phenotypes such as biofilm formation, motility, acid tolerance, virulence, or persister level (for review see Yim et al., 2007; Bernier and Surette, 2013; Andersson and Hughes, 2014). Expression of virulence genes of *L. monocytogenes* may be affected by antibiotics. Ampicillin, vancomycin, and gentamicin reduced the expression of the PrfA-dpendent gene *hly*, encoding listeriolysin O, in *L. monocytogenes* (Nichterlein et al., 1996, 1997), whereas, we found that ampicillin increased expression of another PrfA-dependent gene *inlA*, encoding Internalin A (Knudsen et al., 2012).

The expression of the alternative sigma factor σ^B^-dependent genes is altered by several antibiotics as well as sublethal concentrations of the bacteriocin, pediocin, in *L. monocytogenes* EGD (Shin et al., 2010; Knudsen et al., 2012; Laursen et al., 2015) consistent with induction of the alternative sigma factor σ^S^ in Gram-negative bacteria by antibiotics (Andersson and Hughes, 2014).

Despite the studies cited above little is known about the genetic and physiological response to antibiotics in *L. monocytogenes*. The purpose of the present study was to determine the global transcriptomic response of *L. monocytogenes* EGD to sublethal concentrations of antibiotics, as this is the key to understanding the antibiotics response and any genetic and/or physiological changes that can subsequently lead to antibiotic resistance development of *L. monocytogenes*.

## Materials and Methods

### Bacterial Strains and Growth Conditions

*Listeria monocytogenes* EGD wild-type was used in the study (**Table 1**). Bacterial stock cultures were stored at -80°C and inoculated on Brain Heart Infusion (BHI; Oxoid CM 1135) agar and grown at 37°C overnight. An overnight culture was obtained by inoculating one colony in 5 ml BHI broth and incubating aerobically at 37°C with shaking (250 rpm).

**Table 1:** Bacterial strains and plasmids used in this study.

Strain or plasmids	Genotype and relevant characteristics	Source or reference
***Listeria* strains**	
EGD	*L. monocytogenes* virulent wild-type, MLST ST35	W. Goebel
Δ*lmo1634*	Inframe deletion of *lmo1634*	This study
Δ*lmo1179*	Inframe deletion of *lmo1179*	This study
Δ*lmo1634/*Δ*lmo1179*	Inframe deletion of *lmo1634* and *lmo1179*	This study
Δ*lmaDCBA*	Inframe deletion of *lmaDCBA*	This study
**Plasmids**		
pAUL-A	Temperature sensitive origin of replication, *lacZa’* multiple cloning site, erythromycin resistance marker	Chakraborty et al., 1992
pAUL-A_lmo1634		This study
pAUL-A_lmo1179		This study
pAUL-A_lmaDCBA		This study

### Growth with Sublethal Antibiotic Concentrations

To obtain balanced growth, an overnight culture was diluted 10^9^-fold in 5 ml BHI and grown for 16 h at 37°C at 250 rpm. This culture (OD_600_ < 0.6) was used for inoculation of 50 ml prewarmed BHI to OD_600_ = 0.01 and grown at 37°C with 250 rpm to OD_600_ = 0.1. The culture was split in five by diluting 8 ml culture with 42 ml pre-warmed BHI broth. At OD_600_ = 0.1, ampicillin (0.03 μg ml^-1^; dissolved in sterile MilliQ water, Sigma, A9518), tetracycline (0.035 μg ml^-1^; dissolved in sterile MilliQ water, Sigma, T3383), gentamicin (0.3 μg ml^-1^; dissolved in sterile MilliQ water, Sigma, G3632), or co-trimoxazole (0.2 μg ml^-1^; one part trimethoprim dissolved in sterile MilliQ with 1% glacial acetic acid, Sigma, 92131, and five part sulfamethoxazole dissolved in acetone, Fluka S7507) or sterile MilliQ as control were added. Growth was followed by measuring OD_600_ and bacterial counts were performed a selected time points. Three biological replicates were performed.

### RNA Purification

Samples for RNA sequencing were harvested from antibiotic-exposed and control cells at 0 and 3 h and were quenched for 30 min in ice-cold 2% phenol, 38% ethanol, 62% water, to stabilize RNA (Eriksson et al., 2003). Cells were pelleted by centrifugation and stored at -80°C until total RNA extraction using Trizol (Invitrogen) according to Gómez-Lozano et al. (2012). Total RNA quality and quantity were assessed by an Agilent 2100 Bioanalyser using an RNA 6000 Nano chip and Nanodrop spectrophotometer, respectively.

### Generation of RNA Sequencing Libraries and Sequencing

To reduce the amount of rRNA reads for the RNA sequencing, we isolated mRNA from good quality total RNA (RIN 9.8-10) using the MICROB*Express* kit (Ambion AM1905) according to the manufacture’s protocol. Depletion of the 16S and 23S was verified on a RNA 6000 Nanochip (2100 Bioanalyser, Agilent) and quantity was measured using a Qubit RNA assay (Invitrogen). From each sample, 325 ng mRNA was mixed with 13 μl Elute, Prime and Fragment mix (TruSeq RNA Sample Preparation kit v2, Illumina) and fragmented at 94°C for 8 min prior to proceeding with first strand cDNA synthesis and the TruSeq RNA Sample Preparation kit v2 protocol according to the manufactures protocol (Illumina RS-122-2001 and RS-122-2002). Due to pooling of the samples, the following indices were used: 1-4, 6-16, and 18-21. The quality of the RNA sequencing libraries was investigated by a DNA 1000 assay (2100 Bioanalyser, Agilent). Two peaks were observed, one at 270 bp and one at 1500 bp, the 270 bp peak was isolated using E-gel Size Select 2% (Invitrogen G661002) based on a suggestion from Illumina technical support (Personal communication). The quality of these libraries were controlled using High Sensitivity DNA assay (2100 Bioanalyser, Agilent) and the quantity was assessed using Qubit dsDNA HS assay (Invitrogen). The libraries were pooled using equal amount of pmol to generate a final pooled sample with a concentration of 10.3 nM. This pooled sample was sequenced using HI2000 Sequencing to generate paired-end reads of 100 bp by BGI Hong Kong. Libraries from three independent biological replicates of each treatment were generated except gentamicin of which one biological replicate was omitted due to low quality of the library generated.

### Analysis of RNA Sequencing Data

RNA sequencing data were imported into and analyzed using CLC Genomic workbench (CLC Aarhus, Denmark version 6.0) as described (Laursen et al., 2015). In brief, due to non-normal nucleotide distribution, reads were trimmed by removing the 15 first nucleotides from the 5′ end. To verify the use of the EGDe genome (NC_003210.1) as the reference, we performed a SNP analysis comparing the trimmed reads with the published EGD (MLST ST12; Bécavin et al., 2014) and EGDe genome (MLST ST35; Glaser et al., 2001) with a threshold of 85% variant frequency. Six SNPs with three causing amino acid changes located in *lmo2121, lmo0184*, and *rsbV* genes were detected when comparing our RNA sequencing data to the EGDe reference genome (data not shown). In contrast, there were 25,237 SNPs detected between our EGD and the EGD strain recently published (MLST ST12; Bécavin et al., 2014). Therefore the strain used in the present study is similar to the EGDe strain, originally published by Glaser et al. (2001) and this genome sequence was used as reference genome for the RNA sequencing analysis. sRNAs published by Wurtzel et al. (2012) were included in the reference genome. Reads Per Kilobase per Million (RPKM) was used as the expression value and gene expression of antibiotic-exposed samples were compared with MilliQ control within the same time point. For evaluation of significance, a statistical filter was used including a *t-*test *p* < 0.05 with Baggerly’s test, multiple testing correction *q*-value < 0.05 and a twofold cut-off using only data from two biological replicates due to the loss of the third gentamicin replicate. The gene expression data has been deposited in the NCBI Gene Expression Omnibus and are accessible through GEO accessions number GSE65558. Genes that were significantly differentially expressed were assigned into functional categories based on cluster of orthologous groups (COGs) of *L. monocytogenes* EGDe genes^1^. Hypergeometric distribution test was performed in Excel and a significance level of *p* = 0.01 was used to identify COGs that were overrepresented.

### Verification in Gene Expression Using Quantitative Reverse Transcription PCR (qRT-PCR)

The expression of eight genes was verified by qRT-PCR as described (Knudsen et al., 2012). In brief, one biological replicate used for RNA sequencing and one new biological replicate was used as input for generation of cDNA using Superscript III reverse transcriptase (Invitrogen). 2× SYBR Green PCR Master Mix (Applied Biosystems) was used for qRT-PCR and reactions were run on Mx3000P (Stratagene) on the following program: one cycle at 95°C for 10 min, followed by 40 cycles at 95°C for 30 s and 60°C for 1 min followed by a dissociation curve. Water was included as non-template controls (NTCs) and positive control consisted of genomic DNA from *L. monocytogenes* EGD. Primers (Supplementary Table S1) were either previously published or designed using Primer3^2^. Expression levels were normalized using the geometric mean of the *rpoB* and 16S rRNA housekeeping genes, and calculated using the comparative Ct method (2^-ΔΔCt^; Schmittgen and Livak, 2008).

### Killing Kinetics

We determined the degree of bactericidal/bacteriostatic activity of the four antibiotics by standard killing kinetic experiments as previously described (Knudsen et al., 2013). In brief, an overnight culture was diluted 10^6^-fold and grown for 16 h at 37°C at 250 rpm to obtain a standardized stationary phase culture. This 16 h culture was diluted to OD_600_ = 0.1 with BHI broth and 2 ml of OD_600_ = 0.1 culture was treated as follows: ampicillin (0.03, 3, and 30 μg/ml), tetracycline (0.035, 3.5, and 35 μg/ml), gentamicin (0.3, 15, and 30 μg/ml) and co-trimoxazole (0.2, 10, and 20 μg/ml) being the concentration used in the RNA seq experiment, 100 and 1000X the concentration for the bacteriostatic antibiotic and 50 and 100X the concentration for the bactericidal antibiotic. Cultures were incubated at 37°C at 250 rpm during antibiotic treatment. Bacterial counts were determined just before treatment and at subsequent time points by plate counting on BHI agar. The experiment was performed with three independent biological replicates. When investigating the susceptibility of the mutants, the protocol was slightly modified to be able to detect changes in amount of killing. The 16 h culture was diluted to OD_600_ = 0.4, and treated with 3.0 μg/ml ampicillin, 3.5 μg/ml tetracycline, 30 μg/ml gentamicin, and 10 μg/ml co-trimoxazole with two or three biological replicates.

### Constructions of Mutants

Gene splicing by overlap extension (gene SOEing) was used to create a recombinant gene fragment for an in-frame deletion mutant in the *lmo1179, lmo1634*, and *lmaDCBA* (Horton et al., 1990). Primers (Supplementary Table S1) were constructed on the basis of the published sequence of *L. monocytogenes* EGD-e (Glaser et al., 2001). Chromosomal DNA and plasmid extractions, restriction enzyme digestions and DNA ligations were performed according to standard protocols (Sambrook et al., 1989). The SOEing fragment for each mutant was cloned into pAUL-A (Chakraborty et al., 1992) and the plasmids harboring the SOEing fragment were isolated and verified by sequencing. The generation of the deletion mutant was performed as described by Guzman et al. (1995). Presumptive mutants were verified by PCR and sequencing by GATC (Köln, Germany) or Macrogen (Amsterdam, Netherlands).

### Biofilm Formation

The effect of sublethal antibiotic concentrations on biofilm formation was investigated using a modified O’Toole and Kolter (1998) assay. In brief, an overnight culture was diluted 1:100 in BHI with or without antibiotics and 100 μl were inoculated into wells of 96-well microtitre plates (U96 MicroWell^TM^ Plates, Nunc, #163320) with eight technical replicates. Microtitre plates were incubated for 24 h at 37°C. The biomass as planktonic cells was measured at OD_600_ before crystal violet staining. The amount of biofilm formation was measured by staining with 1% crystal violet for 15 min, followed by washing and the crystal violet-stained biofilm was dissolved in 96% ethanol. After 30 min, 100 μl was transferred to a new microtitre plate and absorbance at 590 nm was determined (Labsystems Multiskan RC or SpectraMax i3, Molecular Devices). The experiment was performed with three biological replicates.

### Statistical Analysis

Bacterial counts and OD_600_ measurements from each biological replicate were log_10_ transformed before statistical analysis using the macro, Analysis ToolPak, in Microsoft Excel. *F*-test was used to test for equal Variances of the sample populations and Student’s *t*-test with equal or unequal variance was used when appropriate with a significance level of *p* < 0.05.

## Results

To investigate the impact of sublethal antibiotic concentrations on gene expression in *L. monocytogenes* EGD, we analyzed the global transcriptome by RNA sequencing after exposure of the bacterium to ampicillin, tetracycline, gentamicin, or co-trimoxazole. Ampicillin and gentamicin were selected as they are the first choice of antibiotics for treating listeriosis. Tetracycline and co-trimoxazole were included as they caused an ‘all up’ or ‘all down’ expression of selected virulence and σ^B^-dependent genes in a previous study (Knudsen et al., 2012). Collectively, the antibiotics also represent all three major classes of antibiotics being inhibitors of cell wall, protein and DNA synthesis. Two of the antibiotics (ampicillin and tetracycline) are bacteriostatic to *L. monocytogenes* and two (gentamicin and co-trimoxazole) are bactericidal (Hof et al., 1997; Hof, 2003; Knudsen et al., 2013).

*Listeria monocytogenes* EGD was exposed for 3 h to an antibiotic concentration that caused a slight increase in doubling time (approximately 10%; Supplementary Figure S1). We have previously found that shorter exposure time and lower concentrations to an antimicrobial, i.e., pediocin had very little effect on gene expression (Laursen et al., 2015). We anticipated that the combination of concentration and exposure time would allow us to investigate the secondary response to antibiotics causing phenotypic changes.

**FIGURE 1:**
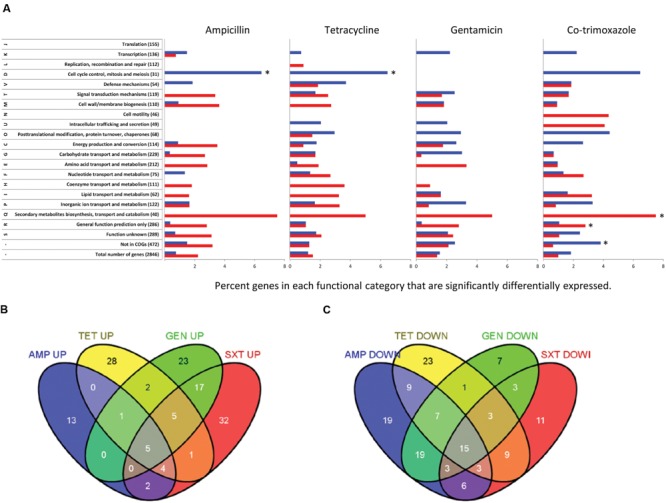
***Listeria monocytogenes* EGD genes differentially expressed when exposed to ampicillin, tetracycline, gentamicin, or co-trimoxazole. (A)** Functional categories of differentially expressed genes in response to the four antibiotics. Genes that passed the statistical filtering (*p* < 0.05, *q* < 0.05 and a twofold cut-off) are shown as percentage of genes in each functional category up-regulated (blue) or down-regulated (red), respectively, when comparing antibiotic-exposed *L. monocytogenes* EGD with MilliQ control. The list of genes included in each functional category was based on cluster of orthologous groups (COGs) of *L. monocytogenes* EGD-e genes (http://www.ncbi.nlm.nih.gov/sutils/coxik.cgi?gi=204%25253e). Asterisk indicate that the functional category was overrepresented in a hypergeometric distribution test (*p* = 0.01). **(B)** Venn diagram with up-regulated genes showing antibiotic-specific and common genes significantly differentially expressed genes. **(C)** Venn diagram with down-regulated genes showing antibiotic-specific and common genes significantly differentially expressed genes. Ampicillin (AMP), tetracycline (TET), gentamicin (GEN), and co-trimoxazole (SXT).

Each of the four antibiotics caused between 106 and 119 genes or sRNA to be differentially expressed as compared to a non-treated control when using a statistical filter of *p* < 0.05, *q* < 0.05 and a threshold of twofold (Supplementary Tables S2 and S3) corresponding to 3.6–4.0% of all genes and sRNA. A hypergeometric distribution test of the functional category analysis showed that only five groups were overrepresented (**Figure 1A**). The ‘cell cycle control, cell division, and chromosome partitioning’ category was overrepresented among genes upregulated by ‘ampicillin and tetracycline. Secondary metabolites biosynthesis, transport and catabolism’ and ‘general function prediction only’ were overrepresented among genes repressed by co-trimoxazole and ‘not in COG’ was overrepresented in genes upregulated by co-trimoxazole.

### Common Differential Gene Expression Caused by All Four Antibiotics

Twenty genes were affected in a similar manner by all four antibiotics, with five and 15 genes being up- and down-regulated, respectively (**Figures 1B,C**). Ten of the 15 genes repressed by all four antibiotics were σ^B^-dependent (Kazmierczak et al., 2003; Hain et al., 2008; Raengpradub et al., 2008; Oliver et al., 2009) and a further 57 other σ^B^ dependent genes were repressed by one, two, or three of the antibiotics indicating that 3 h exposure to antibiotics unexpectedly repressed the general stress response (Supplementary Table S4). The 10 σ^B^-dependent genes repressed by all four antibiotics included the *opuCABCD* operon encoding a betaine-carnitine-choline ABC transporter and two LPXTG peptidoglycan binding protein (*inlH* and *lmo0880*). Two of the five non-σ^B^-dependent repressed genes were *alsS* and *lmo1992* (Supplementary Table S4) encoding an alpha-acetolactate synthase and alpha-acetolactate decarboxylase. These genes convert pyruvate to acetoin indicating lower production of acetoin in antibiotic-exposed cells. The third of the five genes was *pyrR* that encodes a regulator of the pyrimidine biosynthesis and has uracil phosphoribosyltransferase activity. Tetracycline and co-trimoxazole cause repression of *pyrR* higher than 3.8-fold leading to repression of the full downstream operon (*lmo1839-31*) encoding for pyrimidine biosynthesis. Gentamicin caused a 2.3-fold reduction of *pyrR* leading to down-regulation of *pyrPBC* (*lmo1839-7*). *pyrR* was 2.0-fold repressed by ampicillin and the pyrimidine biosynthesis pathway was not significantly differentially expressed demonstrating that a certain threshold of *pyrR* expression is needed to cause a significant repression of the full operon. Only one of three genes (*lmo1885*) involved in the purine biosynthesis was also differential expressed by tetracycline and co-trimoxazole.

Five genes were induced by all four antibiotics (**Figure 1B** and Supplementary Table S4). The expression of *lmo1634* was the most or second most upregulated gene by all four antibiotics with induction levels between 5.5- and 35.6-fold. *lmo1634* encodes a bifunctional acetaldehyde-CoA/alcohol dehydrogenase (ADH) containing both an ADH and an acetaldehyde dehydrogase (ALDH) as well as a NAD^+^ and Fe^2+^ binding domains and converts acetyl-CoA to ethanol (Jagadeesan et al., 2010). High expression of *lmo1634* (>32-fold) correlated with significant induction of both *lmo2105* and *lmo2104* (>3.1-fold) by the two bactericidal antibiotics, gentamicin and co-trimoxazole. In contrast, the two bacteriostatic antibiotics only induced the expression of *lmo1634* 8.2-fold or less, and the expression of *lmo2105* and *lmo2104* were also lower (between 2.0 and 2.2-fold), although the *p*-value of *lmo2104* when exposed to tetracycline was 0.0617, i.e., above our statistical filter. *lmo2105* (*feoB*) encodes the ferrous iron transport protein B, which is co-transcribed with *lmo2104 (feoA)* encoding ferrous iron transport protein A.

### Antibiotic-Specific Gene Expression

One hundred and six genes were differentially expressed in response to ampicillin. Of these, 31 mRNA and sRNA genes (29.2%) were only affected by ampicillin. Twelve genes were uniquely induced by ampicillin and nine of these are controlled by CesRK, LisRK, or LiaSR (Nielsen et al., 2012).

Of the 111 genes differentially expressed by tetracycline, 51 (45.9%) genes were specific for tetracycline. The target of tetracycline is the 30S subunit of the ribosome (Grayson et al., 2010), and the low tetracycline concentration uniquely induced expression of several ribosomal subunits of both 30S and 50S, i.e., *rplK-rplA, rplS, rpsP, rspA*, and *rpmE2.* Expression of *inlAB* and *inlH* encoding Internalin A, B, and H were repressed consistent with our previous study (Knudsen et al., 2012).

Hundred and sixteen genes were differentially expressed by gentamicin and 23 and seven of these genes were specifically induced or repressed, respectively. Among other gentamicin specifically induced *clpB* encoding an Clp ATPase, which could indicate an accumulation of misfolded protein in the cytosol.

Although, both tetracycline and gentamicin are protein synthesis inhibitors and both act on 30S rRNA, only three differentially expressed genes were shared for the two antibiotics indicating differential physiological response. *lmo1138* and *clpE* were induced by both antibiotics and encode a Clp ATP-dependent proteases and an AAA+ ATPase chaperone, respectively, which similar to *clpB* induction by gentamicin indicate an accumulation of misfolded protein.

Forty three genes and sRNA were uniquely expressed by co-trimoxazole and of these, 32 were induced and 11 repressed. Carbonic anhydrase that is the target sulfonamides such as sulfamethoxazole (Capasso and Supuran, 2014) and *lmo0811* encoding a carbonic anhydrase was 3.5-fold decreased by co-trimoxazole. Co-trimoxazole also affected expression of several metabolic genes including *gap, pflA, pdhA*, and *ctaB.* Thus, along with the common transcriptional shift from acetoin production to acetaldehyde and ethanol caused by all four antibiotics, co-trimoxazole appeared to cause further effects on the central metabolism as compared to the other antibiotics.

### Three Antibiotics Cause Opposite Changes in Gene Expression Pattern

A locus of 15 genes encoding two operons was induced by co-trimoxazole and repressed by ampicillin and gentamicin (Supplementary Table S5). This locus, *lmaDCBA* and the downstream *lmo0119-lmo0129*, is the monocin locus encoding an incomplete cryptic prophage. The *lmaDCBA* operon encodes the *L. monocytogenes* antigen A-D which has a temperature-dependent expression and is important for virulence (Schäferkordt and Chakraborty, 1997; Hain et al., 2012), however, antibiotic-dependent expression has not been described previously.

### Summary of Transcriptomic Analysis

Three different expression profiles were observed. All four antibiotics altered expression of a small number of genes leading to a common expression pattern causing a common repression of the σ^B^-dependent genes, but also a shift in metabolism from production of acetoin to ethanol. Secondly, the antibiotic-specific expression pattern included genes indicative of target and/or mode of action of the different antibiotics. Thirdly, the monocin locus showed an antibiotic-dependent expression pattern with induction by co-trimoxazole and repression by gentamicin and ampicillin.

To verify the RNA sequencing data, qRT-PCR was performed with eight selected representing genes that are differentially expressed (up or down) and genes with no difference in gene expression. The genes include *opuCA, lmo1634* and *lmaA*, then used to compare fold changes obtained by the two different methods (Supplementary Figure S2). The correlation coefficient between the two methods was 0.93 indicating a good correlation between the differential gene expression observed by RNA sequencing and by qRT-PCR.

### Verification of the Bactericidal Action of the Four Antibiotics

**FIGURE 2:**
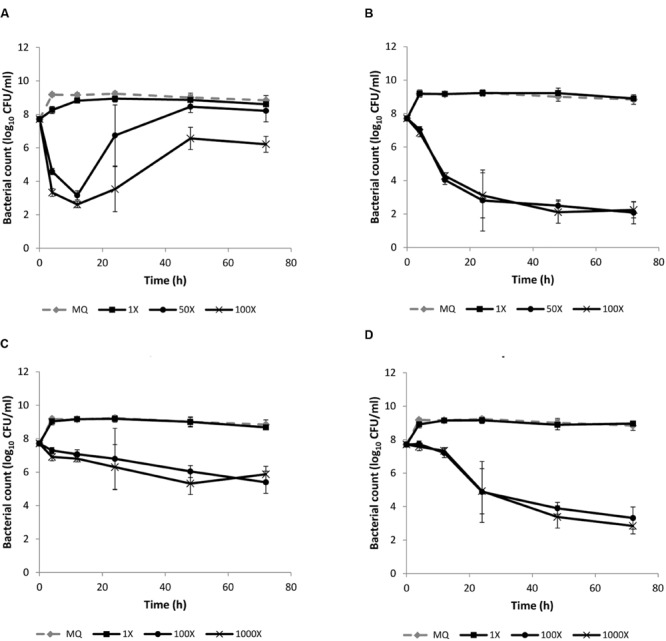
**Killing of *L. monocytogenes* EGD by **(A)** gentamicin, **(B)** co-trimoxazole, **(C)** tetracycline, and **(D)** ampicillin.** An early stationary phase culture (16 h at 37°C) was diluted to OD_600_ = 0.1 and exposed to either MilliQ or different concentrations of the four antibiotics. The concentrations are given relative to the concentration of antibiotic used for the transcriptomic analysis (1X), i.e., 50X (for gentamicin and co-trimoxacole), 100X (for all four antibiotics), and 1000X (for ampicillin and tetracycline). The experiment was performed with three biological replicates and error bar are standard deviation.

We assessed the quantitative differences in bactericidal/bacteriostatic action of the four antibiotics to investigate if there was a relationship between the degree of shift in metabolic gene expression and the level of bactericidal action (Supplementary Figure S3). We tested 100-fold higher concentrations than used in the transcriptomic analyses. Gentamicin and co-trimoxazole caused a 5 and 3.4-log reduction in bacterial count after 12 h (**Figures 2A,B**), respectively, whereas, ampicillin and tetracycline caused less than 1-log reduction after 12 h (**Figures 2C,D**). After 72 h the reduction in bacterial count was 2.3-log for tetracycline and more than 4-log for ampicillin, indicating a bactericidal action of ampicillin after prolonged treatment. Thus the level of killing by the four antibiotics after 12 h reflected the degree of *lmo1634*-expression at 3 h exposure to sublethal concentration of antibiotics.

Ampicillin is normally considered bacteriostatic against *L. monocytogenes* based on minimal inhibitory concentration (MIC) determinations (Hof et al., 1997; Hof, 2003), however, similar to our study Appleman et al. (1991) found a bactericidal action with longer exposure time (48 h) and consistent with ampicillin being first and best choice of antibiotics for treatment of listeriosis (Hof et al., 1997; Kvistholm Jensen et al., 2010; Cossart, 2011).

Regrowth was observed after killing with gentamicin (**Figure 2A**) and colonies from these CFU enumerations were not resistant to gentamicin (data not shown), indicating an induced gentamicin tolerance during the killing experiment.

To investigate the hypothesized relationship between the bactericidal action and metabolic shift, we made several attempts to quantify acetoin and ethanol first by HPLC and secondly by GC–MS. Unfortunately, components of the growth substrate, BHI, interfered with the HPLC measurements. We used acetone extraction combined with GC–MS for quantification of acetoin and ethanol but low concentrations and other factors such as sampling order caused to high standard deviations although a trend being supportive of the hypothesis was observed.

### Deletion of the Aldehyde Oxidoreductase *lmo1179* Led to Antibiotic Tolerance

**FIGURE 3:**
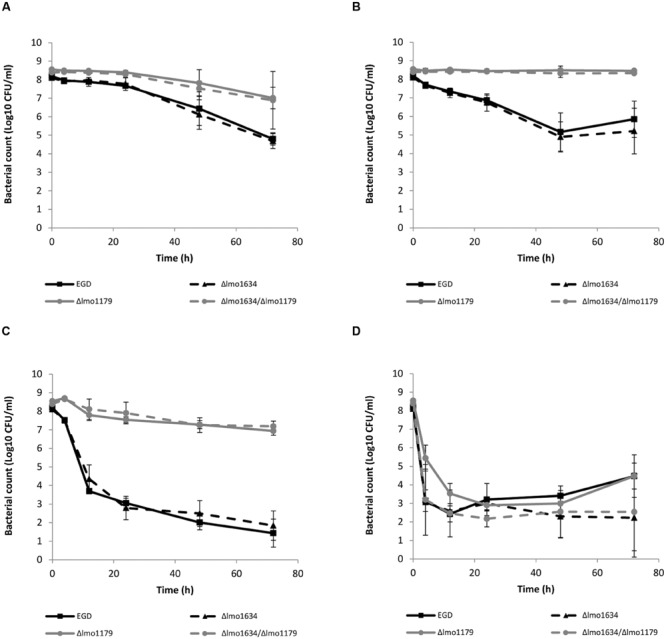
**Killing of *L. monocytogenes* wild type EGD (■, black full line), Δ*lmo1634* (▲, black broken line), Δ*lmo1179* (●, gray full line) and Δ*lmo1634*/Δ*lmo1179* (●, gray broken line) mutants with ampicillin **(A)**, tetracycline **(B)**, co-trimoxazole **(C)**, or gentamicin **(D)** at 37°C.** An early stationary phase culture (16 h) was diluted to OD_600_ = 0.4 and exposed to 3 μg/ml ampicillin **(A)**, 3.5 μg/ml tetracycline **(B)**, 10 μg/ml co-trimoxazole **(C)**, or 30 μg/ml gentamicin **(D)**. The experiment was performed with three biological replicates and error bar are standard deviation.

The induction levels of *lmo1634* during antibiotic exposure, led us to hypothesize that *lmo1634* is playing a role in antibiotic tolerance by causing increased production of ethanol which is normally only produced under anaerobic conditions (Romick et al., 1996). We expected the Δ*lmo1634* mutant to have altered antibiotic susceptibility when compared to the wild type EGD, however, this was not the case (*p* = 0.18–0.72 at 72 h; **Figures 3A–D**). We speculated this could be due to redundancy between *lmo1634* and other genes with the same function. Three other genes are annotated as ADH genes in the genome of *L. monocytogenes* EGD-e of which two are of different protein families (*lmo0773* and *lmo2836*). A blastp show high homology (E-value 2e^-129^ and 44% identity over 52% coverage) between the third annotated ADH (*lmo1179* or *eutE*) and the ALDH domain of *lmo1634* and more likely encode an aldehyde oxidoreductase. The ADH domain of *lmo1634* has three homologies (*eutG* or *lmo1171, lmo1166*, and *lmo1165*) with E-values between 4e^-44^ and 2e^-88^ (between 31–41% identity with 45–46% coverage). These four genes (*lmo1165, lmo1166, lmo1171*, and *lmo1179*) are all located in the locus encoding the vitamin B12 biosynthesis genes and utilization of propanediol and ethanolamine. With several possible genes being redundant of the ADHs, we deleted the *lmo1179* to force the mutant to reroute to ethanol production by either of the other ADHs. Both the Δ*lmo1179* mutant and the Δ*lmo1634/*Δ*lmo1179* double mutant were indeed more tolerant to killing with high concentration of ampicillin (*p* = 0.004 and 0.08 at 72 h, respectively; **Figure 3A**), tetracycline (*p* = 0.043 and 0.047 at 72 h; **Figure 3B**) and co-trimoxazole (both *p* = 0.0002 at 72 h; **Figure 3C**) but not to gentamicin (*p* = 0.99 and 0.23 at 72 h, respectively; **Figure 3D**). A similar pattern were observed when grown in a MIC growth assay where MIC was twofold higher for the Δ*lmo1179* mutant and the Δ*lmo1634/*Δ*lmo1179* double mutant (data not shown) when grown with tetracycline and co-trimoxazole. Both the single and double mutant grew more poorly than the wild type with a generation times of 131 and 149 min, respectively, compared to the wildtype at 103 min when grown in BHI broth without antibiotics at 37°C (Supplementary Figure S4).

### Antibiotic-Dependent Biofilm Formation in *L. monocytogenes* EGD Correlates with Monocin Expression

**FIGURE 4:**
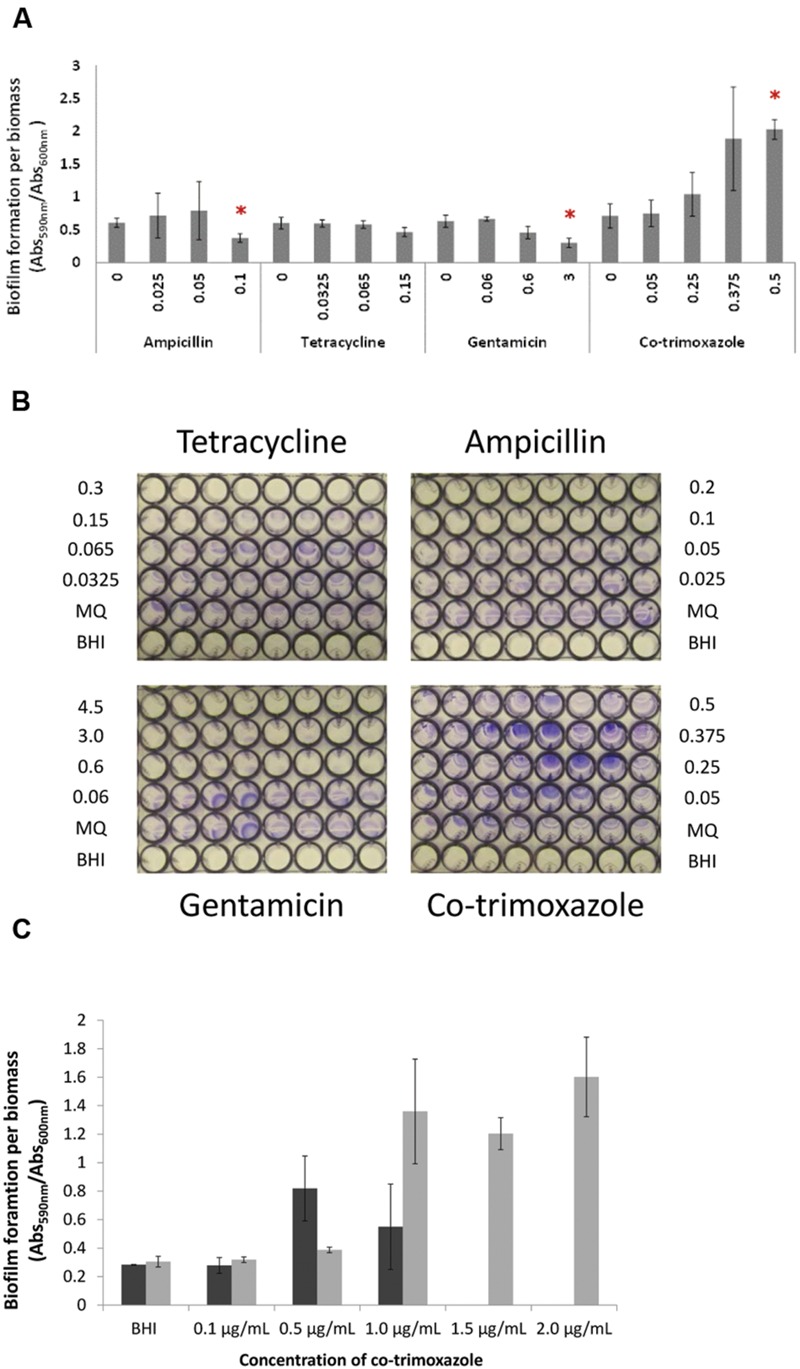
**The effect of sublethal antibiotic concentrations on biofilm formation of *L. monocytogenes* EGD at 37°C. (A)** Biofilm formation of wild type EGD being exposed to increasing concentration of antibiotics measured as crystal violet stained biofilm and measured spectrometrically at 590 nm and calibrated to biomass (measured as planktonic cells at OD_600__nm_). Asterisk denote *p* < 0.05 when comparing biofilm of the control to the antibiotic exposed biofilm. **(B)** Images of crystal violet stained biofilm formed by wild type EGD being exposed to increasing concentration of antibiotics. Eight technical replicates were performed at each experiment. Representative images are shown. **(C)** Biofilm formation of wild type (black bar) and *lmaDCBA* mutant (gray bar) with increasing concentration of co-trimoxazole ranging from 0.1 to 2 μg/ml co-trimoxazole. The experiment was performed with two biological replicates and error bar are standard deviation.

Biofilm formation is affected by antibiotics in several bacteria (Bernier and Surette, 2013; Nguyen et al., 2014), and we investigated if the four antibiotics affected biofilm formation in *L. monocytogenes* EGD. We calibrated all biofilm to biomass measured as planktonic cells (OD_600_) to ensure that effects on growth/maximum cell density were not affecting the results. Sublethal concentrations of tetracycline did not affect the biofilm formation/biomass (*p* = 0.107; **Figures 4A,B**). Increasing co-trimoxazole concentrations significantly increased the biofilm formation per biomass at 0.5 μg/ml (*p* = 0.001) whereas ampicillin and gentamicin significantly reduced the biofilm formation per biomass (*p* = 0.012 and 0.008, respectively). These changes in biofilm formation correlated with expression of the cryptic prophage locus including *lmaDCBA* operon as co-trimoxazole increased expression of the monocin locus and ampicillin and gentamicin repressed the expression of the locus as measured by RNA seq and qRT-PCR of planktonic cells. The monocin locus encodes a cryptic prophage that produces a listeriolytic tail and we hypothesized that increased expression of the monocin locus of planktonic cells would increase monocin production and cell lysis thus increasing biofilm formation. We attempted to determine the level of monocins in sterile supernatant but no plaques were observed in a standard phage-plaque assay indicating that the monocin concentration in the supernatant was too low to kill indicator strains (data not shown).

### The Cryptic Prophage, Monocin, Is Involved Antibiotic-Dependent Killing and Biofilm Formation

**FIGURE 5:**
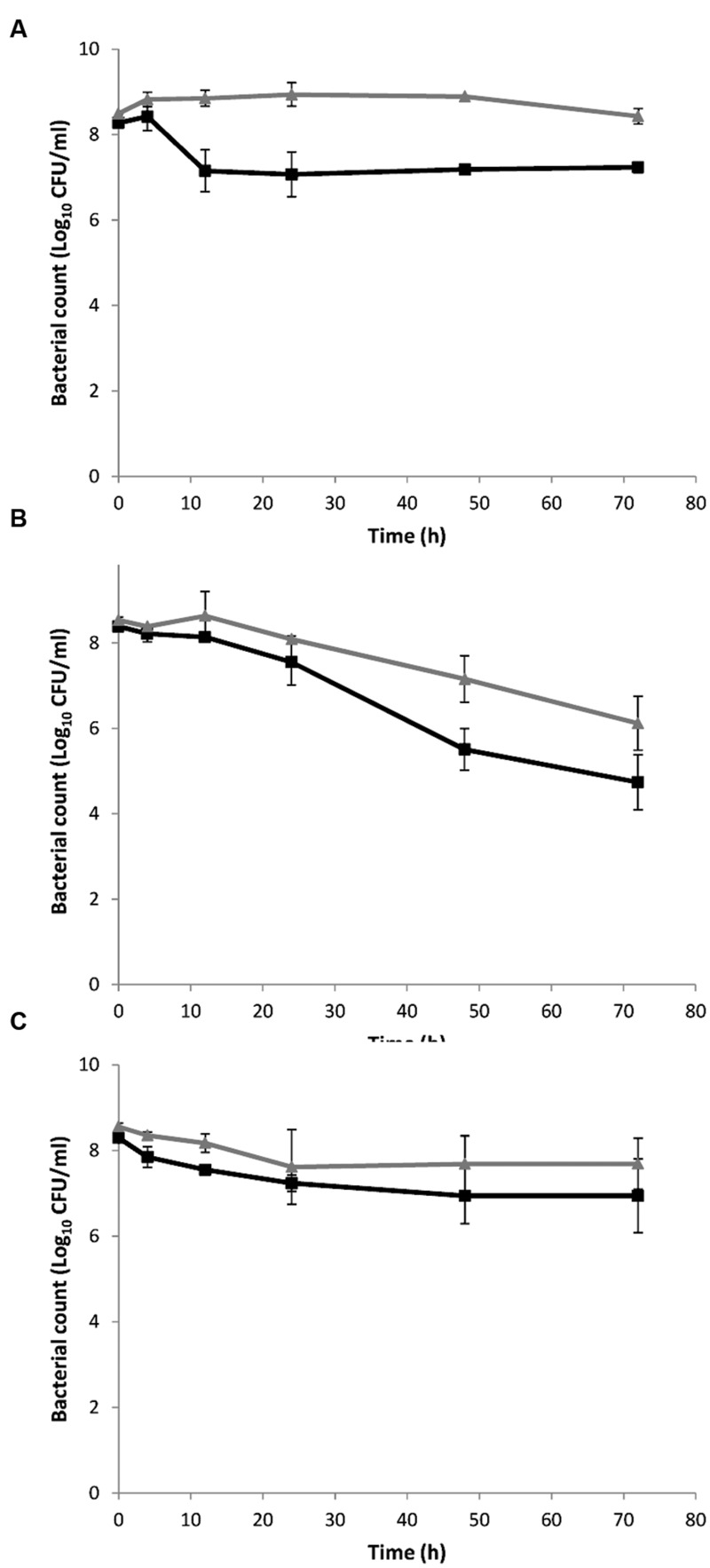
**Killing of *L. monocytogenes* wild type EGD (■) and the Δ*lmaDCBA* (▲) mutant with co-trimoxazole **(A)**, ampicillin **(B)**, and tetracycline **(C)** at 37°C.** An early stationary phase culture (16 h) was diluted to OD_600_ = 0.4 and exposed to 10 μg/ml co-trimoxazole **(A)**, 3 μg/ml ampicillin, **(B)** and 3.5 μg/ml tetracycline **(C)**. The experiment was performed with two biological replicates, except ampicillin that was performed with three biological replicates, and error bar are standard deviation.

To investigate the presumed correlation between monocin expression of planktonic cells and the antibiotic-dependent biofilm formation, we constructed a Δ*lmaDCBA* mutant. In a killing assay, the Δ*lmaDCBA* mutant was significantly (*p* < 0.046) more tolerant by killing of co-trimoxazole at 12 h and afterward (**Figure 5A**) as well as killing by ampicillin after 48 and 72 h (*p* = 0.009 and *p* = 0.031, respectively; **Figure 5B**) but not when treated with tetracycline (*p*-values ranging from 0.055 to 0.61; **Figure 5C**) indicating a role of monocin in antibiotic killing of planktonic cells. Secondly, we investigated the role of monocin in biofilm formation when exposed to increasing concentration of co-trimoxazole and, consistent with increased survival during killing with co-trimoxazole, the Δ*lmaDCBA* mutant grew with higher concentration of co-trimoxazole than the wild type (Supplementary Figure S5A). Similar to the wild type having increased biofilm formation per biomass with increasing growth-inhibiting co-trimoxazole concentration, the Δ*lmaDCBA* mutant also had increased biofilm formation (Supplementary Figure S5B) and biofilm formation per biomass (**Figure 4C**) indicating that that the cryptic prophage, monocin, is not involved in co-trimoxazole-dependent biofilm formation (**Figure 4C**).

## Discussion

In this study, we demonstrate that sublethal concentrations of several antibiotics affect both gene expression and physiology in *L. monocytogenes* EGD. Three distinct profiles of expression changes were observed: (i) a common response to all four antibiotics, (ii) an antibiotic-specific response indicative of the antibiotic mode of action and/or target, and (iii) a differential response of the monocin locus that was dependent on the type of antibiotic. We speculate that these different responses of *L. monocytogenes* EGD to antibiotics in separate ways led the bacterium to induce genes that are involved in antibiotic tolerance. In general, the data support that although a slight growth reduction with an approximately 10% increase in doubling time was caused by the antibiotics, the antibiotic effect was sufficient to elicit a transcriptomic response.

**FIGURE 6:**
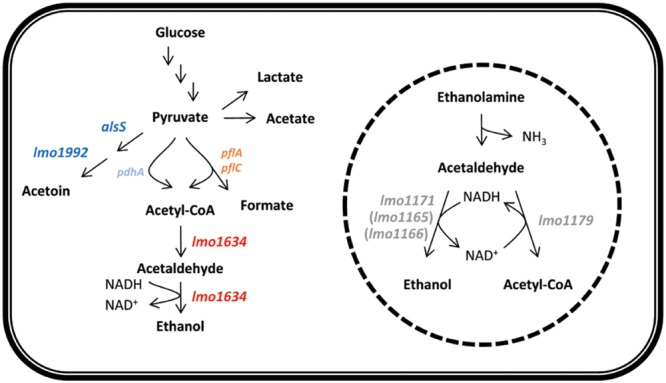
**Model of the central metabolism indicating the shift from acetoin to ethanol production observed by all four antibiotics.** Blue indicate decreased expression of *lmo1992* and *alsS* and red indicate increased expression of *lmo1634* by all four antibiotics. Pyruvate formate-lyase encoded by *pflAC* (light red) and involved in degradation of pyruvate under anaerobic condition is induced by gentamicin and co-trimoxazole but not ampicillin and tetracycline whereas pyruvate dehydrogenase encoded by *pdhA* (light blue) involved in degradation of pyruvate under aerobic condition are down-regulated by co-trimoxazole but not any of the other antibiotics. Broken circle illustrate the microcompartment in which ethanolamine is degraded and the degradation pathway of ethanolamine to ethanol and acetyl-CoA. Neither of the four genes *lmo1179, lmo1171, lmo1165*, or *lmo1166* (gray) are differentially expressed by any of the four antibiotics.

*Listeria monocytogenes* responded to antibiotics by remodeling the gene expression of the central metabolism, which indicate a shift from acetoin to ethanol production driven by a shift in expression of mainly *alsS, lmo1992, lmo1634*, and *lmo2104-5*. This shift correlate with a shift from aerobic to anaerobic metabolism as *L. monocytogenes* under aerobic conditions converts 26% of the carbon from glucose to acetoin, while no acetoin is produced under anaerobic conditions (Romick et al., 1996). In contrast, ethanol is specifically produced under anaerobic conditions (Romick et al., 1996). This shift in metabolic gene expression was more pronounced for the bactericidal antibiotic than the bacteriostatic compounds. It was recently shown that *Burkholderia thailandensis* tolerate antibiotics by adapting to and inducing anaerobic nitrate respiration and the tolerance could be eliminated by oxygenating the system or with the addition of nitrate (Hemsley et al., 2014). Fumarate is the likely alternative electron acceptor in *L. monocytogenes* under anaerobic conditions (Müller-Herbst et al., 2014) and consistent with *B. thailandensis*, gentamicin and co-trimoxazole, but not ampicillin and tetracycline, induced *lmo0355*, encoding a fumarate reductase, more than fourfold at 3 h supporting our hypothesis with a larger metabolic shift for cell exposed to bactericidal antibiotic than to bacteriostatic. Based on the transcriptomic analysis and functional analysis of the *lmo1634* gene encoding a bifunctional acetaldehyde-CoA/ADH, we speculate that this gene plays a central role in the metabolic shift (**Figure 6**). The bifunctional acetaldehyde-CoA/ADH Lmo1634 has 49% protein identity (E-value 0.0 over 872 amino acids) to AdhE of *Escherichia coli* but in *L. monocytogenes* EGD-e this protein is also known as LAP (*Listeria* adhesion protein) and promotes bacterial adhesion to enterocyte-like Caco-2 cells (Jagadeesan et al., 2010). Both *lmo1634* and *adhE* are induced by anaerobic conditions (Leonardo et al., 1996; Burkholder et al., 2009) and in *E. coli*, AdhE also acts as a H_2_O_2_ scavenger under aerobic conditions and has an important role in protection against H_2_O_2_ stress (Echave et al., 2003). The role of Lmo1634 in oxidative stress in *L. monocytogenes* is unknown but both 3 mM ferricyanide (FeCN) and 6 mM dithiothreitol (DTT) that altered redox potential repress the expression of *lmo1634* (Ignatova et al., 2013). However, a Δ*lmo1634* mutant was as sensitive to antibiotic as the wild type EGD whereas a mutant in *lmo1179* (*eutE*) was highly tolerant to ampicillin, tetracycline and co-trimoxazole, but not gentamicin. *lmo1179* (*eutE*) are located in the *eut* operon that involved in utilization of ethanolamine and encoded an aldehyde oxidoreductase that convert acetaldehyde to acetyl-CoA under generation of NADH from NAD^+^ (Garsin, 2010; Mellin et al., 2014). Ethanolamine is a degradation product of phosphatidylethanolamine that is abundant in mammalian and bacterial cell membranes (Garsin, 2010) and the *eut* genes involved in virulence in *L. monocytogenes* (Joseph et al., 2006; Mellin et al., 2014). In this study, we show that the absence of *lmo1179* (*eutE*) cause antibiotic tolerance to three of four antibiotic tested and we speculate this tolerance is caused by rerouting of all acetaldehyde to ethanol, i.e., anaerobic metabolism and that there is redundancy among the ADHs in *L. monocytogenes*. However, interesting neither of the *eut* genes are significantly affected by any of the four antibiotics.

We suggest that *L. monocytogenes* EGD shift to anaerobic condition and production of ethanol to avoid respiration. During aerobic respiration, NADH are re-oxidized to NAD^+^ and generation of reactive oxygen species (ROS) during aerobic respiration are speculated to be involved in killing (Kohanski et al., 2007; Dwyer et al., 2014), however, this role of ROS in antibiotic killing is debated (Keren et al., 2013; Liu and Imlay, 2013). In line with Liu and Imlay (2013), we have previously shown that it is not likely that *L. monocytogenes* EGD-e generate ROS during killing as oxidative stress mutants were not more sensitive to bactericidal antibiotic than the wild type (Feld et al., 2012). Based on this study, we speculate that ethanol production by bifunctional acetaldehyde-CoA/ADH containing both NAD^+^ and Fe^2+^ binding domains and *lmo1634* is induced under antibiotic exposure to eliminate recycling of NADH to NAD^+^ by respiration that potentially could lead to production of ROS. Ma et al. (2015) recently showed that when *E. coli* is treated with vancomycin or cefotaxime iron is accumulated, however, under iron starvation the level of killing was increased and instead ROS was accumulated. *L. monocytogenes* co-induced the ferrous iron transporter FeoAB encoded by *lmo2105-04* with *lmo1634* and deletion of *feoAB* in *Bacteroides fragilis* lead to metronidazole resistance (Veeranagouda et al., 2014). Whereas deletion of *fri* in *L. monocytogenes* encoding a non-heme, iron binding ferritin-like protein (Fri) cause the mutant to have increased sensitivity to penicillin G (Krawczyk-Balska et al., 2012).

Interestingly, the metabolic shift seems to be slightly different when treated with gentamicin, as the Δ*lmo1179* mutant was sensitive to gentamicin as the wild type. Gentamicin is assumed to be transported over the membrane by proton-motive force (PMF) generated when the electron transport chain oxidizes NADH to NAD^+^ (Taber et al., 1987). Thus *L. monocytogenes* by induction of anaerobiosis would block for transport of gentamicin and induce tolerance as observed when treating with gentamicin (**Figure 2A**). However, the rerouting for higher ethanol production was not capable of inducing tolerance when treated with gentamicin in the *lmo1179* mutant indicating that the metabolic shift is more complex than just a shift to ethanol production. Indication of the complexity is also observed for co-trimoxazole that had the highest fold change of metabolic genes and also induced other metabolic genes such as *pflA* and *pflBC* genes encoding the pyruvate formate-lyase and pyruvate formate-lyase activating enzyme and repression of *pdhA* encoding a subunit of pyruvate dehydrogenase but still supportive of the shift to anaerobic metabolism (Wang Q. et al., 2010). However, sulfa drugs such as sulfamethoxazole and trimethoprim (the two component of co-trimoxazole) are also described as antimetabolite drugs as they are inhibitors of different metabolic enzymes such as carbonic anhydrases (Capasso and Supuran, 2014). In summary, we hypothesize that *L. monocytogenes* shift to ethanol production that eliminate NADH and anaerobiosis to adapt to the sublethal concentration and subsequently prepare for lethal conditions however the driver of the metabolic remodeling in *L. monocytogenes* remains unknown and further investigation are necessary. Interestingly, when *L. monocytogenes* was exposed to alkaline conditions it induced a similar shift to anaerobic metabolism changing from acetoin to ethanol production (Nilsson et al., 2013).

Prophages often are present in bacterial genomes (Fortier and Sekulovic, 2013) and are important for biofilm formation, antibiotic tolerance, and UV-resistance (Qin et al., 2007; Selva et al., 2009; Carrolo et al., 2010; Wang X. et al., 2010; Rabinovich et al., 2012; Martínez-Garcia et al., 2015). All lineages and strains of *L. monocytogenes* investigated contain the monocin locus that encodes a cryptic prophage and whilst its general role is not known, it is expressed intracellularly in murine macrophages and is important for virulence in mice (Schäferkordt and Chakraborty, 1997; Hain et al., 2012). The antibiotic-dependent expression of monocin in planktonic cells correlated with biofilm formation at 37°C, i.e., induced by co-trimoxazole and repressed by ampicillin and gentamicin at 3 h and could indicate that bactericidal antibiotics cause production of listeriolytic bacteriophages-tail-like particles. Lytic monocin particle can be induced by UV-light likely caused by DNA damage (Zink et al., 1995) and we speculated that the co-trimoxazole cause similar kind of DNA damage. We constructed a Δ*lmaDCBA* mutant, which according to the published data should be equivalent to a full monocin mutant (Wurtzel et al., 2012), and found that in the absence of the monocin it was more tolerant against both co-trimoxazole and ampicillin likely indicating production of lytic tail particles and thereby cell lysis. We speculated that lysis of a *L. monocytogenes-*subpopulation by monocin will release extracellular DNA, which is an important component of *L. monocytogenes* biofilm and initiates the initial attachment (Harmsen et al., 2010). It is phage-dependent lysis of a subpopulation of *Streptococcus pneumonia* and *Staphylococcus epidermidis* that provide eDNA for biofilm formation (Qin et al., 2007; Carrolo et al., 2010), and in *S. epidermidis* and *S. aureus* this eDNA release is mediated by the autolysins (Qin et al., 2007; Kaplan et al., 2012). *L. monocytogenes* encodes an autolysin in the monocin locus, *lmo0129*, which is indeed 3.4-fold upregulated by co-trimoxazole (Supplementary Table S3). However, similar to the wild type EGD, the Δ*lmaDCBA* mutant had increased biofilm formation with increasing concentration of co-trimoxazole suggesting that monocin not involved in this biofilm phenotype and the increased expression of monocin is only important in planktonic cells. However in summary, consistent with other bacteria such as *Pseudomonas putida* (Martínez-Garcia et al., 2015), in the absence of cryptic prophages mutants are more tolerant stressors such as antibiotic stress and UV shown to be caused by the lytic phage particle and indicate that killing can be obtained by controlling the bacteriophage expression.

Research on antibiotics has predominantly focused on their antimicrobial activity and the ability of bacteria to develop resistance. However, during the last decade, the possible role of antibiotics as signals or cues affecting bacterial physiology has become an area of intensive research. It has been suggested that antibiotics have a metabolic role in the antibiotic-producing organism as biosynthesis of antibiotics such as phenazine eliminates excess reducing power and alter the intracellular redox state (Wang Y. et al., 2010; Andersson and Hughes, 2014). However, the main hypothesis in this area is that low concentration of antibiotic can modulate gene expression on the target organism, consistent with this study, and act as signals, cues or coercion for gene expression causing phenotypic changes (for review see Yim et al., 2007; Bernier and Surette, 2013; Andersson and Hughes, 2014). This study indicates that sublethal antibiotic concentrations induce genes that prime *L. monocytogenes* to alter its physiology that may allow it to tolerate higher lethal concentrations and thereby survive. Thus we interpret response as beneficial and a cue for gene expression and physiological changes for *L. monocytogenes*. Recent data indicate that not only *L. monocytogenes* but also other pathogens respond to sublethal antibiotic concentrations as cues in different ways and some have adapted different strategies to prime for antibiotic survival. Consistent with our results, *B. thailandensis* tolerate antibiotics by adapting to anaerobic metabolism (Hemsley et al., 2014), whereas *M. tuberculosis* induce a specific dormancy regulon for growth arrest and antibiotic survival (Baek et al., 2011; Koul et al., 2014) and ciprofloxacin induce persister formation via the TisB toxin in *E. coli* (Dörr et al., 2010). We have previously shown that exposure of *L. monocytogenes* to pediocin induce genes involved in resistance of pediocin (Laursen et al., 2015). Interestingly, Seyedsayamdost (2014) recently showed that sublethal concentrations of trimetroprim that is a component of co-trimoxazole induce silent biosynthetic gene clusters for drug production in *B. thailandensis* indicating an alternative survival strategy by production an antibiotic to kill the competitor in the environment.

In summary, we show that sublethal concentrations of antibiotic can be a specific cue that causes differential gene expression leading to assumed beneficial alternations in physiology, such as a shift to anaerobic metabolism, induction of monocin expression and biofilm formation of *L. monocytogenes*, which could prime for survival when treated with lethal concentration of antibiotics.

## Author Contributions

GK and LG contributed the conception of this study; GK and LG designed the experiments; GK, AF, and YN performed the experiments; GK and AF analyzed data; GK and LG interpreted data; GK and LG drafted the manuscript; and GK, AF, YN, and LG approved the manuscript.

## Conflict of Interest Statement

The authors declare that the research was conducted in the absence of any commercial or financial relationships that could be construed as a potential conflict of interest.

## Footnotes

1 http://www.ncbi.nlm.nih.gov/sutils/coxik.cgi?gi=204%25253e

2 http://frodo.wi.mit.edu/primer3/input.htm
