# Supplementary material for: Sublethal Concentrations of Antibiotics Cause Shift to Anaerobic Metabolism in Listeria monocytogenes and Induce Phenotypes Linked to Antibiotic Tolerance
Source: Front Microbiol. 2016 Jul 12;7:1091. doi: 10.3389/fmicb.2016.01091 (PMC4940397; doi:10.3389/fmicb.2016.01091)
Supplement: Supplementary file 1 [file Data_Sheet_1.PDF]

Supplementary Material

Sublethal concentrations of antibiotics cause shift to anaerobic metabolism in *Listeria monocytogenes* and induce phenotypes linked to antibiotic tolerance

Gitte M. Knudsen, Arvid Fromberg, Yin Ng and Lone Gram

*** Correspondence: Lone Gram, gram@bio.dtu.dk**

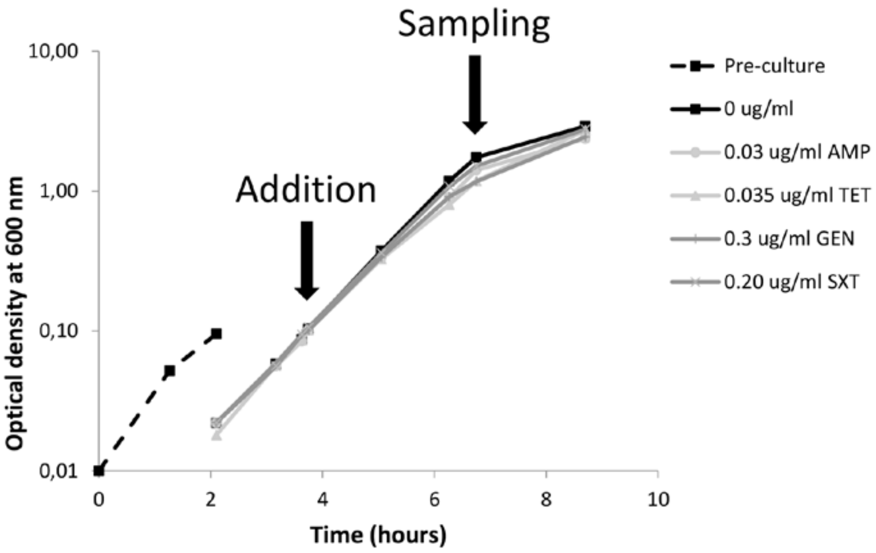

Fig. S1. Growth kinetics of *L. monocytogenes* EGD showing pre-culturing with a dilution step to obtain five identical cultures and time points for addition of antibiotics (AMP for ampicillin, TET for tetracycline, GEN for gentamicin and SXT for co-trimoxazole) or MilliQ as control as well as time point of sampling for transcriptomic analysis. Growth was measured by OD_{600}. The experiment was performed with three biological replicates and one representative is shown.

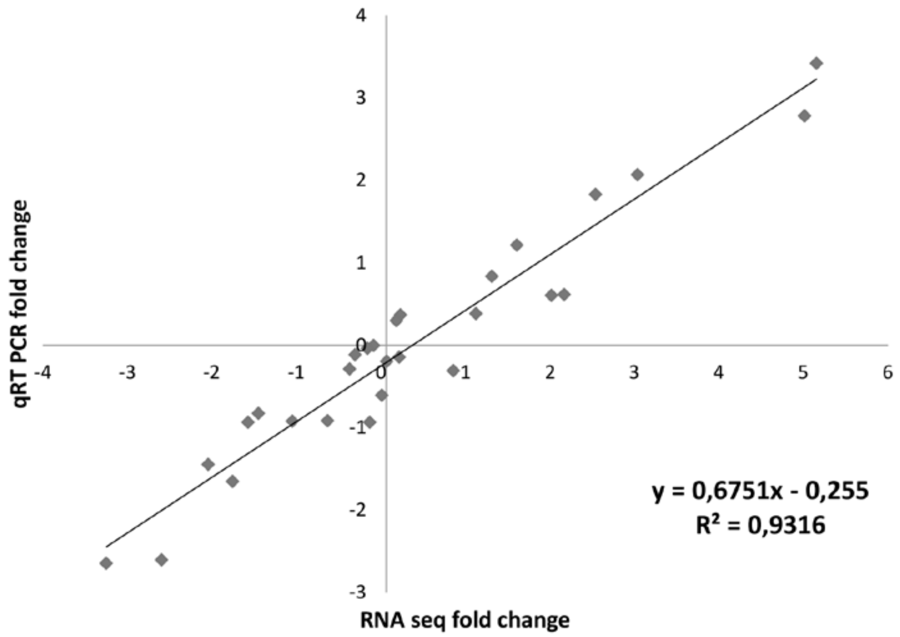

Fig. S2. Verification of the RNA sequencing data of *Listeria monocytogenes* exposed to ampicillin, tetracycline, gentamicin or co-trimoxazole was performed using RT-qPCR using one biological replicate used for RNA Sequencing and one newly produced biological replicate.

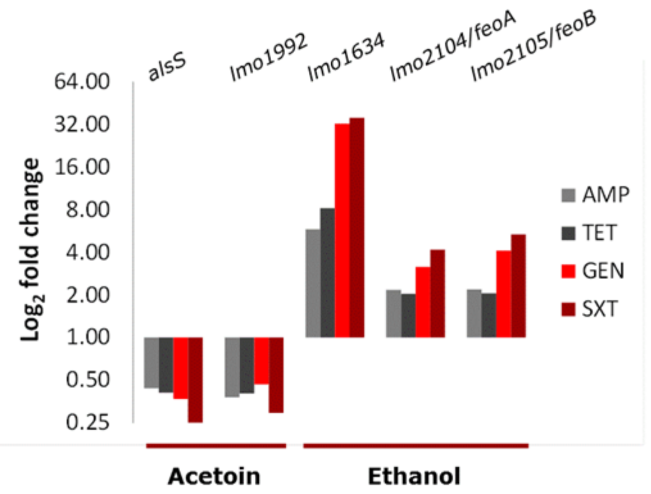

Fig. S3. All four antibiotics affect expression of *Listeria monocytogenes* metabolic genes. Correlation of RNA sequencing data of metabolic genes with bactericidal antibiotic – gentamicin (orange bar) and co-trimoxazole (red bar) – and the bacteriostatic antibiotics – ampicillin (grey bar) and tetracycline (black bar).

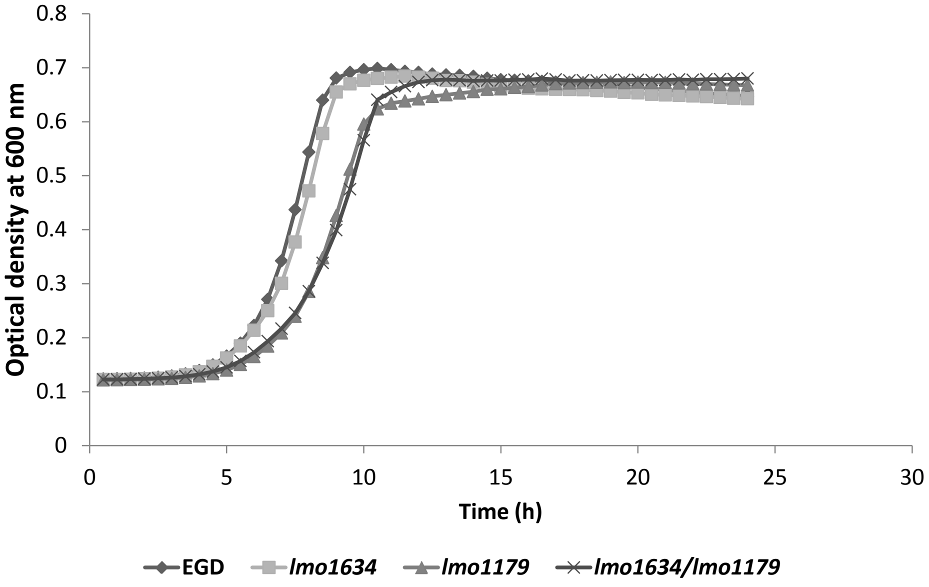

Fig. S4. Growth of the wild type EGD (♦), Δ*lmo1634* (▪), Δ*lmo1179* (▲) and Δ*lmo1634*/Δ*lmo1179* (×) mutants in BHI broth over 24h at 37°C. Growth was performed in microtiter plates and measured by SpectraMax i3x Multi-Mode microplate reader. Graph is an average of three biological replicates each with two technical replicates.

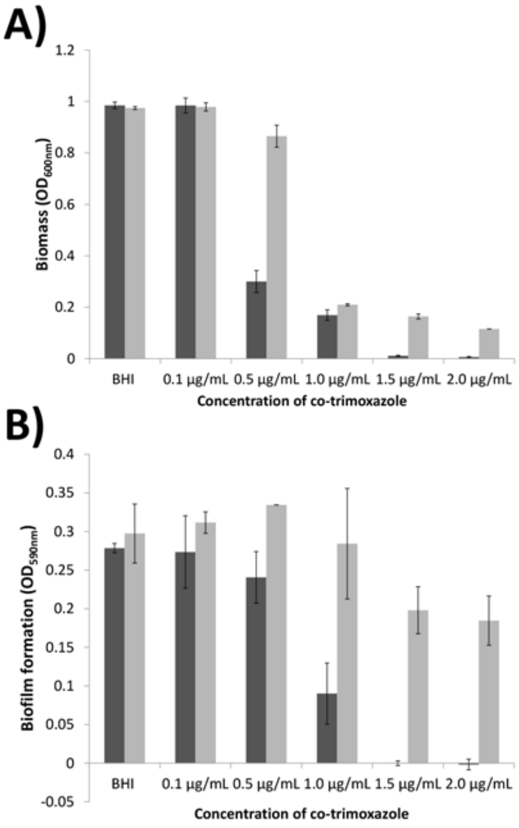

Fig. S5. Biofilm formation of *Listeria monocytogenes* wild type and Δ*lmaDCBA* mutant with increasing concentration of co-trimoxazole ranging from 0.1 to 2 µg/ml co-trimoxazole. A) Biomass measured at OD_{600} and B) Biofilm formation measured by crystal violet staining at OD_{590}. The experiment was performed with two biological replicates and error bars are standard deviations.

Table S1. Sequence of primers used in this study for verification of the transcriptomic analyses of *Listeria monocytogenes* exposed to sublethal concentrations of antibiotics by qRT-PCR and construction of deletion mutants.

Name	Sequence	Reference
Mutant construction and promoter fusion		
lmo1634_SOEA_hind	CCATCTAAAA**AAGCTT**TCACTTCAGAAAAAGCATGC[1]	This study
lmo1634_SOEB	TGCCATTTTCCAGAACCTCC	This study
lmo1634_SOEC	*GGAGGTTCTGGAAAATGGCA*TGAAACTAGCTAACTTGTCC[2]	This study
lmo1634_SOED_Bam	TCTGCCTCGG**GGATCC**AATTCACCGCAAGTGCTAGG	This study
lmo1634_up_CLC	CATGCTACTTACTCTTCTTC	This study
lmo1634_down_CLC	CGCCCTAAGTCAACCAACAT	This study
lmo2365_SOEA_Hin	ATGC**AAGCTT**GACTCAAAATCCTGTGCC	This study
lmo2365_SOEB	AAGCATAACTACCCTCTCC	This study
lmo2365_SOEC	*GGAGAGGGTAGTTATGCTT*AAAAGGTGGAGCTGGAG	This study
lmo2365_SOED_Bam	ATCG**GGATCC**AATACAAATTTGCACCGG	This study
lmo2365_up2	CTAAACAACGCCCACATCCT	This study
lmo2365_down2	GCCCAGTTAATGCGGAAGTA	This study
lmo2365_deletion	CGGAGTAACTGAGCGGAAAG	This study
lmaDCBA_SOEA_Xba	TTTTCTGCTA**TCTAGA**CTTGATTTAAGCCTCGTTCC	This study
lmaDCBA_SOEB	ATCCATCCATTCCCACTTCC	This study
lmaDCBA_SOEC	*GGAAGTGGGAATGGATGGAT*GAAACACCTGTAGCACCAGC	This study
lmaDCBA_SOED_Bam	AAATCATGGT**GGATCC**CACTATCCACTTCTTCGTCC	This study
lmaDCBA_up	GCTAATAGTTTGGC	This study
lmaDCBA_down	GATGTTAATTCCACCG	This study
lmaDCBA_deletion	TGCACATCTGATTTGGTTCTTT	This study
1179_AHind	GGGG**AAGCTT**GCTATCGAAGCAGGAAAAGCG	This study
1179_B	ACGAACTTCTTGGATTGAGCG	
1179_C2	CGCTCAATCCAAGAAGTTCGTTGCAGACGCACTGATCCAAAGTT	This study
1179_DBam	GGGG**GGATCC**CTGTTGCTGCTTTAACTGCG	This study

lmo1179_up	AAAAGCAGCGAATGTCG	This study
lmo1179_down	GATAGTAATTCACCAACGC	This study
pUC19.1	ACACTTTATGCTTCCGGCTC	
pUC19.2	TACGCCAGCTGGCGAAAGG	
qRT-PCR		
rpoB-fwd	CGTCGTCTTCGTTCTGTTGG	(van der Veen and Abee 2010)
rpoB-rev	GTTCACGAACCACACGTTCC	(van der Veen and Abee 2010)
16S-rRNA-fwd	GATGCATAGCCGACCTGAGA	(van der Veen and Abee 2010)
16S-rRNA-rev	TGCTCCGTCAGACTTTCGTC	(van der Veen and Abee 2010)
inlA-f	GGTCTCACAAACAGATCTAGACCAAGT	(Sue et al. 2004)
inlA-r	TCAAGTATTCCACTCCATCGATAGATT	(Sue et al. 2004)
ofr2420-f	TGTTGCTTGTATTACGGTTATTGC	(Knudsen et al. 2012)
ofr2420-r	TTGGGCCCTATTTTGATATTTG	(Knudsen et al. 2012)
hfq-f	GGTGGACAAGGGTTACAGGA	(Knudsen et al. 2012)
hfq-r	CAACGCGTCCTCTTAACTGA	(Knudsen et al. 2012)
Lma-f	AAGATACAGTTGGCGGATGG	This study
Lma-r	GAGACTCTGCATCAGCGACA	This study
Lmo2173-f	GATATGCCGACAACGCTTTT	This study
Lmo2173-r	CCAACTGTAAGCGCAGTGAA	This study
Lmo1634-f	CCAAGCTGGCTTCAAAGTTC	This study
Lmo1634-r	GGTATTTGTCGCCAACACCT	This study

1) Bold indicate restriction site
2) Italic indicates sequence complementary part to SOEB primer.

Table S2. Number of *Listeria monocytogenes* genes differentially expressed when an exponentially growing culture was exposed to sublethal concentration of ampicillin, tetracycline, gentamicin or co-trimoxazole for 3h. All genes passed the statistical filtering (p<0.05, q<0.05 and a two-fold cut-off), when comparing antibiotic-exposed *L. monocytogenes* with MilliQ control.

		Number of differentially expressed genes			
	Total number of gene and sRNA	Up-regulated by antibiotic		Down-regulated by antibiotic	
		Genes	sRNA	Genes	sRNA
Ampicillim	106	23	2	79	2
Gentamicin	116	45	1	67	2
Tetracycline	111	51	2	58	0
Co-trimoxazole	119	61	5	50	3

Table S4. List of 20 *Listeria monocytogenes* genes that were either up- or down regulated by all four antibiotics when compared to the MilliQ control. Exponentially growing culture was exposed to sublethal concentrations of antibiotics causing a 10% growth rate reduction and samples for RNA sequencing analysis were taken after 3h. Expression from each antibiotic are normalized to MilliQ control giving fold changes and are average of two biological replicates.

		Baggerley's test: AB 3h vs MQ 3h original values - Weighted proportions fold change					
**Gene**	**Synonym**	**AMP**	**TET**	**GEN**	**SXT**	**Description**	**SigB dependent**
lmo0471	*lmo0471*	2.486	2.284	2.901	2.285	hypothetical protein	
lmo1634	*lmo1634*	5.834	8.220	32.380	35.670	bifunctional acetaldehyde-CoA-alcohol dehydrogenase	
lmo2105	*lmo2105*	2.195	2.055	4.110	5.352	ferrous iron transport protein B	
lmo2410	*lmo2410*	2.363	2.261	4.932	6.637	hypothetical protein	
lmo2686	*lmo2686*	2.988	2.450	3.125	3.999	hypothetical protein	
lmo0019	*lmo0019*	-3.714	-3.474	-2.998	-2.717	hypothetical protein	SigB
lmo0134	*lmo0134*	-3.523	-2.209	-2.527	-2.457	*E. coli* YjdJ protein	SigB
inlH	*lmo0263*	-4.196	-2.920	-2.446	-2.619	internalin H	SigB
lmo0573	*lmo0573*	-2.321	-5.089	-3.791	-4.957	hypothetical protein	
lmo0670	*lmo0670*	-7.495	-2.512	-3.844	-2.882	hypothetical protein	SigB
fliQ	*lmo0677*	-6.111	-3.284	-3.469	-2.715	flagellar biosynthesis protein FliQ	
lmo0880	*lmo0880*	-6.802	-2.815	-4.928	-4.742	wall associated protein precursor LPXTG motif	SigB
opuCD	*lmo1425*	-3.640	-3.117	-2.658	-2.598	betaine-carnitine-choline ABC transporter membrane p	SigB
opuCC	*lmo1426*	-4.139	-3.227	-2.799	-2.683	glycine betaine-carnitine-choline ABC transporter osmoprotectant-binding protein	SigB
opuCB	*lmo1427*	-4.432	-3.408	-2.752	-2.510	glycine betaine-carnitine-choline ABC transporter membrane protein	SigB
opuCA	*lmo1428*	-4.115	-3.368	-2.722	-2.963	glycine betaine-carnitine-choline ABC transporter ATP-binding protein	SigB
pyrR	*lmo1840*	-2.024	-3.812	-2.309	-4.141	bifunctional pyrimidine regulatory protein PyrR uracil phosphoribosyltransferase	

lmo1992	*lmo1992*	-2.282	-2.451	-2.713	-3.990	alpha-acetolactate decarboxylase	
alsS	*lmo2006*	-2.646	-2.476	-2.138	-3.403	acetolactate synthase	
sepA	*lmo2157*	-3.508	-2.208	-2.618	-2.197	hypothetical protein	SigB

Table S5. List of 15 *Listeria monocytogenes* genes that were either upregulated by co-trimoxazole and downregulated by ampicillin and gentamicin when compared to the MilliQ control. Exponentially growing culture was exposed to sublethal concentrations of antibiotics causing a 10% growth rate reduction and samples for RNA sequencing analysis were taken after 3h. Expression from each antibiotic are normalized to MilliQ control giving fold changes and are average of two biological replicates.

		Baggerley's test: AB 3h vs MQ 3h original values - Weighted proportions fold change				
**Gene**	**Synonym**	**AMP**	**TET**	**GEN**	**SXT**	**Description**
lmaC	*lmo0116*	-3.687	1.261	-4.491	3.353	Antigen C
lmaB	*lmo0117*	-7.577	-1.188	-7.392	2.995	antigen B
lmaA	*lmo0118*	-6.027	-1.232	-9.487	3.058	antigen A
lmo0119	*lmo0119*	-14.296	-1.146	-8.085	3.846	hypothetical protein
lmo0120	*lmo0120*	-6.750	-1.319	-5.957	2.976	hypothetical protein
lmo0121	*lmo0121*	-5.563	-1.206	-6.570	3.584	bacteriophage minor tail proteins
lmo0122	*lmo0122*	-4.594	-1.153	-6.018	3.395	phage proteins
lmo0123	*lmo0123*	-4.508	-1.237	-4.965	3.537	protein gp18 from Bacteriophage A118
lmo0124	*lmo0124*	-4.605	-1.260	-11.581	3.783	hypothetical protein
lmo0125	*lmo0125*	-6.134	-1.546	-9.645	2.896	hypothetical protein
lmo0126	*lmo0126*	-5.469	-1.391	-6.020	2.996	hypothetical protein
lmo0127	*lmo0127*	-4.677	-1.266	-5.989	2.608	weakly protein gp20 from Bacteriophage A118
lmo0128	*lmo0128*	-5.703	-1.192	-5.615	2.625	a protein from Bacteriophage phi-105 ORF 45
lmo0129	*lmo0129*	-2.819	1.039	-3.892	3.355	autolysin: N-acetylmuramoyl-L-alanine amidase

References

Knudsen,G.M., Holch,A. and Gram,L. (2012) Subinhibitory concentrations of antibiotics affect stress and virulence gene expression in *Listeria monocytogenes* and cause enhanced stress sensitivity but do not affect Caco-2 cell invasion. *J. Appl. Microbiol.* **113**, 1273-1286.

Sue,D., Fink,D., Wiedmann,M. and Boor,K.J. (2004) s^B-dependent gene induction and expression in *Listeria monocytogenes* during osmotic and acid stress conditions simulating the intestinal environment. *Microbiology* **150**, 3843-3855.

van der Veen,S. and Abee,T. (2010) Importance of SigB for *Listeria monocytogenes* static and continuous-flow biofilm formation and disinfectant resistance. *Applied and Environmental Microbiology* **76**, 7854-7860.
